# Study on sustainable development of pharmaceutical health industry under ecological coordination

**DOI:** 10.3389/fpubh.2023.1117701

**Published:** 2023-02-09

**Authors:** Gan Fu, Zhao Zhao

**Affiliations:** ^1^School of Economics and Management, Tongji University, Shanghai, China; ^2^College of Management and Economics, Tianjin University, Tianjin, China

**Keywords:** ecological coordination, pharmaceutical health industry, supply chain, risk disturbance, sustainability, combined contract

## Abstract

Particularly in the post-pandemic period, where public health emergencies offer a greater risk of supply disruptions, the operational hazards of pharmaceutical supply chains are uncertain. One of the main concerns for businesses is how to handle the risk of supply disruption and take the necessary precautions to lower the chance of loss. Pharmaceutical raw material suppliers, pharmaceutical manufacturers and medical institutions constitute a complete three-tiered supply chain. On the basis of this, in Materials and methods part, a share contract based on buyback proceeds is created as a result, and a combination contract based on centralized decision-making and decentralized decision-making is employed to maximize the order volume of pharmaceutical supply chain participants. An out-of-stock cost pharmaceutical supply chain model is created, and a related solution is provided and measurable examples. In Results and discussion part, to confirm the accuracy of the model and algorithm, numerical examples are employed. Buyback prices and order volumes were subjected to sensitivity analysis, and discussion is had over how various parameters affect a model's performance. Due to supply disruptions, the study's findings show that there is “double sourcing” between upstream pharmaceutical raw materials and downstream major suppliers, necessitating the establishment of a supply chain with numerous standby suppliers. At the same time, modifying the contract parameters can improve the supply motivation of backup suppliers and guarantee the profitability of downstream medical institutions.

## Introduction

The century epidemic and the century of transition that the world is currently experiencing are interwoven, causing instability and uncertainty on a worldwide scale. The global supply chain is forced into the reconstruction stage as a result of geopolitical disputes and numerous trade restrictions (1). The COVID-19 outbreak has caused a severe global shortage of medical supplies, a significant disruption in the pharmaceutical supply chain, uncertainty regarding the fundamental safety of medical staff, and the inability to treat a significant number of affected patients promptly. Governments and pharmaceutical companies, therefore, pay more attention to the risk of supply chain disruption against the backdrop of the increasingly complex and variable external environment and work to adopt targeted strategies to improve their capacity to withstand risks, which is of great significance to ensure the normal functioning of pharmaceutical supply chains (2). This paper examines the risk of supply disruption in the pharmaceutical supply chain. A combination contract model is built based on the study of member income under various decision-making models to investigate how to optimize and coordinate the pharmaceutical supply chain and address the supply interruption issue.

The relevant literature in pharmaceutical supply chain research mainly deals with the mode of operation, risk assessment, and sustainable development. Zhang et al. (3) realized the full life cycle management of medicine by building a monitoring system based on blockchain and deep learning. Salehi et al. (4) used data envelopment analysis (DEA) and fuzzy data envelopment analysis (FDEA) methods to assess pharmaceutical supply chain elasticity. In the risk assessment of the drug distribution supply chain, Zhang et al. (5) discussed multi-attribute decision-making with fuzzy bipolar Hamach correlation mean (BFHCA) operator. The different risks facing green supply chain development in the pharmaceutical industry were empirically analyzed by Kumar et al. (6) and different countermeasures were put forth. Halim et al. (7) constructed the supply chain network of sustainable drugs based on a decision support framework and system and verified and analyzed it with enterprise examples. Liu et al. (8) constructed a system dynamics model considering government dynamic penalties and subsidies for the evolutionary stability strategy of medical device recycling enterprises.

In the research of supply interruption, scholars analyze the supply chain ordering strategy, inventory strategy, and coordination strategy from different perspectives. Olivares-Aguila et al. (9) introduced a system dynamics framework to observe the game behavior of supply chain members and evaluate the impact of supply interruption on supply chain performance. Bo et al. (10) studied the coordinated scheduling problem of multi-product manufacturing supply chain with delivery time constraints under supply chain disruption. Chakraborty et al. (11) while developing mathematical models of SC and SCB proved that retailers are always more willing to take advantage of the capacity advantages of standby suppliers under interrupted supply. Gupta et al. (12) explored the role of early sales strategies in supply chain financing during supply disruptions. Parast also found through empirical data testing that increased R&D investment significantly reduces the impact of environmental and process disruptions on supply chain performance (13). Supply chain contract also plays an important role in supply chain optimization and coordination. Typical supply chain contracts include revenue sharing, volume flexibility, buybacks, and wholesale price contracts (14, 15). Canbulut et al. (16) studied the role of different repo combinations in supply chain performance in a fuzzy demand environment based on credibility theory. Farhat et al. (17) introduced term repo contracts into the supply chain's multi-cycle optimal procurement decisions. Niederhoff et al. (18) reported the supply chain decision behaviors with different attitudes under the wholesale price–revenue sharing contract, and the results showed that the subject's risk preference rendered the revenue-sharing contract invalid. Canbulut et al. (19) believed that the combination of game theory and revenue-sharing contracts can better achieve the optimal equilibrium strategy between suppliers and retailers. Liu et al. (20) introduced the option contract into the supply chain system composed of the government and risk aversion suppliers and derived the optimal reserve decision of government and enterprise emergency supplies. He et al. (21), based on revenue sharing and price subsidy contracts, coordinated the supply chain system to achieve the optimal service integrator scheduling strategy.

Based on the previous literature review, supply disruption research focuses on how to reduce disruption risk, supply chain recovery (22), and risk assessment. There is little research on supply disruption in pharmaceutical supply chain operating scenarios. Meanwhile, it is found that multi-contracts formed by combination can realize supply chain coordination better than single contracts. To this end, a two-stage template of a pharmaceutical supply chain portfolio contract is constructed by introducing revenue-sharing and buyback contracts when supply disruption risk is considered. The reasonable contract parameters are obtained through the simulation of the example, so as to improve the profits of medical institutions and pharmaceutical manufacturers, as well as the supply willingness of backup suppliers, and ensure the stable coordination of the pharmaceutical supply chain.

## Materials and methods

### Model description and assumptions

**Figure 1:**
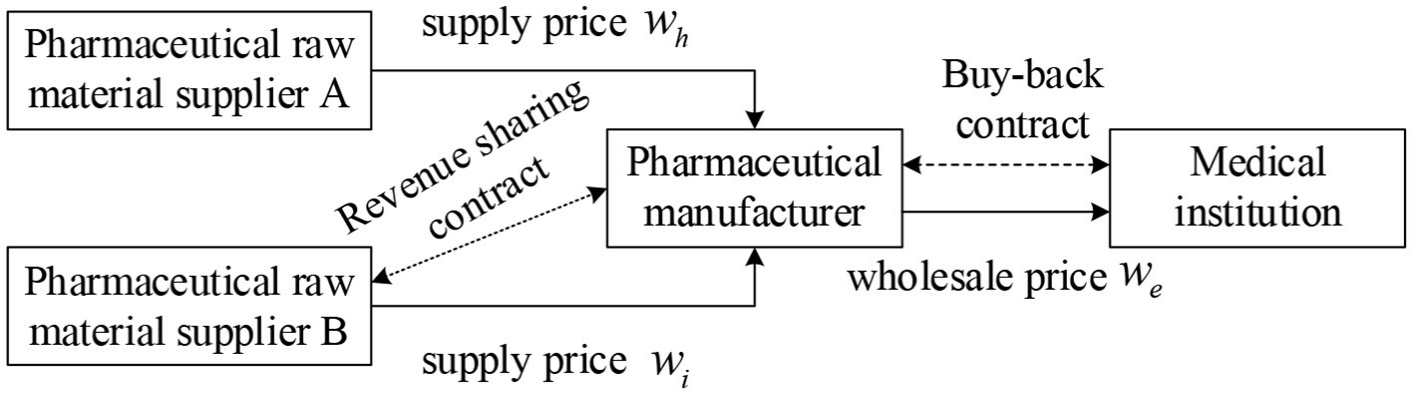
Operation process of the pharmaceutical supply chain under supply interruption.

In this study, we assume that in a three-tiered supply chain consisting of pharmaceutical raw material suppliers, pharmaceutical manufacturers, and medical institutions (Figure 1), there are two pharmaceutical raw material suppliers A and B, which provide the same quality products and services. Among them, the product price of pharmaceutical raw material supplier A is low, but it is prone to supply interruption risk, while the product price of pharmaceutical raw material supplier B is high but stable and reliable. Supplier A is the main supplier of medicine, the manufacturer orders here first, and supplier B is a backup supplier, only when supplier A cannot meet the demand of pharmaceutical manufacturers' orders, pharmaceutical manufacturers will order from supplier B. To improve the enthusiasm of pharmaceutical feedstock supplier B and reduce the shortage loss, the revenue sharing contract is introduced based on dual sourcing to stimulate supplier B to prepare more supplies. In parallel, pharmaceutical manufacturers and downstream medical institutions sign repurchase agreements to ensure the income of medical institutions and improve the overall performance of the pharmaceutical supply chain. The symbols used in the model are shown in Table 1.

**Table 1:** Model symbols and definitions.

**Parameters**	**Definition**
*q*_*h*_	Supplier A's supply to pharmaceutical manufacturer
*q*_*i*_	Supplier B's supply to pharmaceutical manufacturer
*w*_*h*_	Supplier A's supply price to the pharmaceutical manufacturer
*w*_*i*_	Supplier B's supply price to the pharmaceutical manufacturer
*w*_*e*_	Wholesale prices of pharmaceutical manufacturer to medical institution
*c*_*h*_	Unit product cost of supplier A
*c*_*i*_	Unit product cost of supplier B
*p*	Medical sales price of medical institution
*s*	Shortage cost of medical institution
*v*	Residual value of unsold medicine
γ	Price coefficient of pharmaceutical manufacturer' repurchases from medical institution (0 < γ < 1)
κ	Revenue-sharing coefficient
θ	Probability of interruption risk of pharmaceutical backup supplier A(0 < θ < 1)
Q	Order quantity of medical institution

To facilitate the model construction and supply chain coordination strategy analysis, the following hypotheses are proposed:

(1) All players in the pharmaceutical supply chain are fully rational and risk-neutral.

(2) There is *w*_*e*_ > *w*_*i*_ > *w*_*h*_ > *v* to ensure the revenues of pharmaceutical manufacturers and pharmaceutical raw material suppliers A and B.

(3) There is *p* > *w*_*e*_, γ*w*_*e*_ > *v* to ensure the effectiveness of the repurchase contract and the revenue of medical institutions.

(4) Pharmaceutical manufacturers dominate, and pharmaceutical supplier B has sufficient supply capacity to meet the production needs of pharmaceutical manufacturers.

### Construction of two basic models

The methods followed by Wang and Li (22) were used. To obtain the optimal order quantity and maximum revenue of the pharmaceutical supply chain under centralized and decentralized decision-making, a benchmark model is established under centralized as well as decentralized conditions. The evaluation criteria are based on two basic models to judge whether the combination contract can improve the supply capacity of the backup supplier B, reduce the shortage loss of pharmaceutical manufacturers and downstream medical institutions, and realize the optimization and coordination of the whole supply chain.

### Operation of the pharmaceutical supply chain under a centralized decision

When the supply interruption risk occurs for the main supplier A, the total revenue of the pharmaceutical supply chain is:


(1) $$\begin{array}{l} \pi =p\text{ min}\left(q_{i},x\right)+v\text{ max}\left(q_{i}-x,0\right)-s\text{ max}\left(x-q_{i},0\right)-c_{i}q_{i} \end{array}$$

When the main supplier A has no supply interruption risk, the total revenue of the pharmaceutical supply chain is:


(2) $$\begin{array}{l} \pi =p\text{ min}\left(q_{h}+q_{i},x\right)+v\text{ max}\left(q_{h}+q_{i}-x,0\right)\\ \;-s\text{ max}\left(x-q_{h}-q_{i},0\right)-c_{h}q_{h}-c_{i}q_{i} \end{array}$$

Then, the expected revenue function of the pharmaceutical supply chain is:


(3) $$\begin{array}{l} E\left(\pi \right)=\theta [p\text{ min}\left(q_{i},x\right)+v\text{ max}\left(q_{i}-x,0\right)-s\text{ max}\left(x-q_{i},0\right)\\ -c_{i}q_{i}]+\left(1-\theta \right)\\ {}[p\text{ min}\left(q_{h}+q_{i},x\right)+v\text{ max}\left(q_{h}+q_{i}-x,0\right)-s\text{ max}(x-q_{h}\\ -q_{i},0)-c_{h}q_{h}-c_{i}q_{i}] \end{array}$$

The above equation can be rewritten as:


(4) $$\begin{array}{l} \pi =\theta \int_{0}^{q_{i}}\left[px+v\left(q_{i}-x\right)-c_{i}q_{i}\right]f\left(x\right)dx\\ \;+\theta \int_{q_{i}}^{\infty }\left[pq_{i}-s\left(x-q_{i}\right)-c_{i}q_{i}\right]f\left(x\right)dx+\\ 1-\theta \int_{0}^{q_{h}+q_{i}}\left[px+v\left(q_{h}+q_{i}-x\right)-c_{h}q_{h}-c_{i}q_{i}\right]f\left(x\right)dx+\\ \left(1-\theta \right)\int_{q_{h}+q_{i}}^{\infty }\left[p\left(q_{h}+q_{i}\right)-s\left(x-q_{h}-q_{i}\right)-c_{h}q_{h}-c_{i}q_{i}\right]f\left(x\right)dx \end{array}$$

The first and second derivatives of Equation (4) about q_*i*_ can be obtained as:


(5) $$\begin{array}{l} \frac{\partial \pi }{\partial q_{i}}=\left(p-c_{i}+s\right)+\left(v-p-s\right)\left[\theta F\left(q_{i}\right)+\left(1-\theta \right)F\left(q_{h}+q_{i}\right)\right]\\ \frac{\partial^{2}\pi }{\partial {q_{i}}^{2}}=\left(v-p-s\right)\left[\theta f\left(q_{i}\right)+\left(1-\theta \right)f\left(q_{h}+q_{i}\right)\right] \end{array}$$

It is easy to get $\frac{\partial^{2}\pi }{\partial {q_{i}}^{2}}<0$, knowing (4) is a concave function about *q*_*i*_, let $\frac{\partial \pi }{\partial q_{i}}=0$, and get the optimal supply of backup supplier B to meet the conditions:


(6) $$\begin{array}{l} \theta F\left({q_{i}}^{c*}\right)+\left(1-\theta \right)F\left(q_{h}+{q_{i}}^{c*}\right)=\frac{c_{i}-p-+s}{v-p-s} \end{array}$$

Similarly, the second derivative of *q*_*h*_ is <0, and the formula (4) is also a concave function about *q*_*h*_, which has a maximum value. Let $\frac{\partial \pi }{\partial q_{i}}=0,\frac{\partial \pi }{\partial q_{h}}=0$, and the optimal order quantity of the pharmaceutical supply chain under centralized conditions is obtained as:


(7) $$\begin{array}{l} Q_{C}^{*}={q_{h}}^{*}+{q_{i}}^{c*}=F^{-1}\left(\frac{c_{h}-p-s}{v-p-s}\right) \end{array}$$

Analysis of Equation (7) and *F*(*x*) characteristics concluded that the change of supply quantity ${q_{i}}^{c*}$ under centralized decision-making is closely related to the value *v*, that is, the greater the residual value of unsold drugs in pharmaceutical institutions, the greater the backup supplier B can provide more pharmaceutical raw materials; retaining the partial residual value of drugs not only compensates for the procurement losses of pharmaceutical manufacturers but also reduces the supply cost of backup supplier B. Furthermore, we calculated the partial derivative of Equation (5) with respect to θ and got $\frac{\partial^{2}\pi }{\partial q_{i}\partial \theta }>0$. It can be seen that *q*_*i*_ and θ change positively. When the supply interruption probability θ is greater, the supply volume of backup supplier B is greater. At this point, pharmaceutical companies prefer backup suppliers, because the more likely a major supplier A is to be disrupted, the more likely it is that there will be shortages of raw materials produced by pharmaceutical companies. To cope with the shortage loss caused by interruption, pharmaceutical manufacturers are increasingly relying on a more stable raw material backup supplier B to meet the needs of medical institutions and patients.

### Operation of the pharmaceutical supply chain under a decentralized decision

Operation analysis of backup supplier:

To simplify the model calculation, let *q*_*h*_ be a fixed value. Under decentralized conditions, pharmaceutical raw material backup supplier B mainly relies on the classical newsboy model to determine the optimal supply quantity, and its supply capacity model is as follows:


(8) $$\begin{array}{l} L\left(q_{i}\right)=\left(1-\theta \right)E\text{ min}\left[\left(x-q_{h}\right),q_{i}\right]+\theta E\text{ min}\left(x,q_{i}\right)\\ =q_{i}-\theta \int_{0}^{q_{i}}F\left(x\right)dx-\left(1-\theta \right)\int_{q_{h}}^{q_{h}+q_{i}}F\left(x\right)dx \end{array}$$

Under the given production and supply capacity constraints of pharmaceutical raw material backup suppliers, the revenue function is as follows:


(9) $$\begin{array}{l} \pi_{i}\left(q_{i}\right)=w_{i}L\left(q_{i}\right)+\left(1-\theta \right)[\int_{q_{h}}^{q_{h}+q_{i}}v\left(q_{h}+q_{i}-x\right)f\left(x\right)dx\\ -\int_{0}^{q_{h}}\left(c_{i}-v\right)q_{i}f\left(x\right)dx\\ -\int_{q_{h}}^{+\infty }c_{i}q_{i}f\left(x\right)dx]-\theta [\int_{0}^{q_{i}}\left[c_{i}-v\left(q_{i}-x\right)\right]f\left(x\right)dx\\ +\int_{q_{i}}^{+\infty }c_{i}q_{i}f\left(x\right)dx] \end{array}$$

The first and second derivatives of Equation (8) with respect to *q*_*i*_ are obtained as follows:


(10) $$\begin{array}{l} \frac{\partial \pi_{i}\left(q_{i}\right)}{\partial q_{i}}=w_{i}-c_{i}+\left[\left(1-\theta \right)F\left(q_{h}+q_{i}\right)+\theta F\left(q_{i}\right)\right]\left(v-w_{i}\right) \end{array}$$

(11) $$\begin{array}{l} \frac{\partial^{2}\pi_{i}\left(q_{i}\right)}{\partial {q_{i}}^{2}}=\left[\left(1-\theta \right)f\left(q_{h}+q_{i}\right)+\theta f\left(q_{i}\right)\right]\left(v-w_{i}\right)<0 \end{array}$$

(12) $$\begin{array}{l} \left(1-\theta \right)F\left(q_{h}+{q_{i}}^{d*}\right)+\theta F\left({q_{i}}^{d*}\right)=\frac{c_{i}-w_{i}}{v-w_{i}} \end{array}$$

Operation analysis of pharmaceutical manufacturer is as follows:


(13) $$\begin{array}{l} \pi_{e}\left(q_{i}\right)=\left(1-\theta \right)[\int_{0}^{q_{h}+q_{i}}\left[w_{e}x+v\left(q_{h}+q_{i}-x\right)\right]f\left(x\right)dx+\\ \int_{q_{h}+q_{i}}^{+\infty }\left[w_{e}\left(q_{h}+q_{i}\right)-s\left(x-q_{h}-q_{i}\right)\right]f\left(x\right)dx]\\ +\theta [\int_{0}^{q_{i}}\left[w_{e}x+v\left(q_{i}-x\right)f\left(x\right)\right]dx+\\ \int_{q_{i}}^{+\infty }\left[q_{i}w_{e}-s\left(x-q_{i}\right)\right]f\left(x\right)dx]-w_{i}L\left(q_{i}\right) \end{array}$$

Derivation of formula (12) is:


(14) $$\begin{array}{l} \frac{\partial \pi_{e}\left(q_{i}\right)}{\partial q_{i}}=\left[\theta F\left(q_{i}\right)+\left(1-\theta \right)F\left(q_{h}+q_{i}\right)\right]\left(w_{i}-w_{e}-s\right)+w_{e}-w_{i} \end{array}$$

(15) $$\begin{array}{l} \frac{\partial^{2}\pi_{e}\left(q_{i}\right)}{\partial {q_{i}}^{2}}=\left[\theta f\left(q_{i}\right)+\left(1-\theta \right)f\left(q_{h}+q_{i}\right)\right]\left(w_{i}-w_{e}-s\right) \end{array}$$

Let $\frac{\partial \pi_{e}\left(q_{i}\right)}{\partial q_{i}}$ be equal to 0 to get the equation:


(16) $$\begin{array}{l} \theta F\left(q_{i}\right)+\left(1-\theta \right)F\left(q_{h}+q_{i}\right)=\frac{w_{i}-w_{e}}{w_{i}-w_{e}-s} \end{array}$$

According to the *F*(*x*) characteristics, formula (15) is not valid, $\frac{\partial \pi_{e}\left(q_{i}\right)}{\partial q_{i}}>0$ is constant. The revenue function π_*e*_(*q*_*i*_) is a monotone increasing function of the supply quantity *q*_*i*_ of the backup suppliers, which the revenue of the pharmaceutical manufacturers increases with the increase of supply amount.

Operation analysis of medical institution:

Assuming that medical institutions do not sign any contracts with pharmaceutical manufacturers under decentralized decision-making, the expected revenue of medical institutions is:


(17) $$\begin{array}{l} E\left(\pi_{\text{r}}\right)=\theta [p\text{ min}\left(q_{i},x\right)+v\text{ max}\left(q_{i}-x,0\right)-s\text{ max}\left(x-q_{i},0\right)\\ -w_{e}q_{i}]+\left(1-\theta \right)\\ {}[p\text{ min}\left(q_{h}+q_{i},x\right)+v\text{ max}\left(q_{h}+q_{i}-x,0\right)-s\text{ max}\left(x-q_{i}-q_{h},0\right)\\ -w_{e}\left(q_{h}+q_{i}\right)] \end{array}$$

That is, the above formula can be rewritten as:


(18) $$\begin{array}{l} \pi_{r}=\theta [\int_{0}^{q_{i}}\left[px+v\left(q_{i}-x\right)-w_{e}q_{i}\right]f\left(x\right)dx+\int_{q_{i}}^{\infty }[pq_{i}-s\left(x-q_{i}\right)\\ -w_{e}q_{i}]f\left(x\right)dx]+\\ \left(1-\theta \right)[\int_{0}^{q_{h}+q_{i}}\left[px+v\left(q_{h}+q_{i}-x\right)-w_{e}\left(q_{h}+q_{i}\right)\right]f\left(x\right)dx+\\ \int_{q_{h}+q_{i}}^{\infty }\left[p\left(q_{h}+q_{i}\right)-s\left(x-q_{h}-q_{i}\right)-w_{e}\left(q_{h}+q_{i}\right)\right]f\left(x\right)dx] \end{array}$$

According to the same principle of Equation (5), the revenue function π_*r*_ is a concave function *q*_*h*_, *q*_*i*_. Let $\frac{\partial \pi_{r}}{\partial q_{i}}=0,\frac{\partial \pi_{r}}{\partial q_{h}}=0$, the optimal order quantity of medical institutions can be obtained as follows:


(19) $$\begin{array}{l} Q^{*}={q_{h}}^{*}+{q_{i}}^{*}=F^{-1}\left(\frac{w_{e}-p-s}{v-p-s}\right) \end{array}$$

Based on the above analysis, under decentralized decision-making, pharmaceutical manufacturers and medical institutions jointly rely on pharmaceutical main supplier A and backup supplier B to provide raw material products to meet the needs of production and patients. Comparing Equation (16) with Equation (12), it is found that the supply backup supplier B is much larger in centralized decision-making than in decentralized decision-making because backup suppliers usually provide fewer goods in accordance with their own interests, which leads to the failure of the pharmaceutical supply chain to achieve global optimization. Meanwhile, it is found that pharmaceutical manufacturers' revenue is positively correlated with the supply of backup suppliers. When backup suppliers can provide a steady supply of raw materials and products, pharmaceutical companies can further improve their revenues.

### Pharmaceutical supply chain coordination strategy under portfolio contract

In the operation of the pharmaceutical supply chain, major suppliers are at risk of supply disruptions due to cumbersome transportation processes and random factors of substandard raw material quality. At this time, pharmaceutical manufacturers will increase orders to backup supplier B to cope with the loss of production due to shortages of pharmaceutical ingredients. Thus, to realize the smooth operation of the supply chain, upstream enterprises will sign repurchases or revenue-sharing contracts with downstream enterprises, reduce shortage loss caused by supply interruption risk, improve supply enthusiasm of backup suppliers, and realize revenue maximization of each participant.

### Ordering strategy of the medical institution under repurchase contract

The pharmaceutical supply chain has a much higher operational risk than the normal supply. To realize the downstream supply chain optimization and coordination, reduce the storage and sale costs of medical institutions when supply exceeds demand, recover cash flow, and reduce the concerns of large-scale supply, medical institutions will enter repurchase agreements with pharmaceutical companies. There are still two cases of supply interruption and non-interruption, and the following revenue function of medical institutions is obtained:


(20) $$\begin{array}{l} E\left({\pi_{\text{r}}}^{\prime }\right)=\theta [p\text{ min}\left(q_{i},x\right)+\gamma w_{e}\text{ max}\left(q_{i}-x,0\right)-s\text{ max}\left(x-q_{i},0\right)\\ -w_{e}q_{i}]+\left(1-\theta \right)\\ {}[p\text{ min}\left(q_{h}+q_{i},x\right)+\gamma w_{e}\text{ max}\left(q_{h}+q_{i}-x,0\right)\\ -s\text{ max}\left(x-q_{i}-q_{h},0\right)-w_{e}\left(q_{h}+q_{i}\right)] \end{array}$$

The above formula can be rewritten as:


(21) $$\begin{array}{l} {\pi_{r}}^{m}=\theta [\int_{0}^{q_{i}}\left[px+\gamma w_{e}\left(q_{i}-x\right)-w_{e}q_{i}\right]f\left(x\right)dx+\int_{q_{i}}^{\infty }[pq_{i}\\ -s\left(x-q_{i}\right)-w_{e}q_{i}]f\left(x\right)dx]\\ +\left(1-\theta \right)[\int_{0}^{q_{h}+q_{i}}\left[px+\gamma w_{e}\left(q_{h}+q_{i}-x\right)-w_{e}\left(q_{h}+q_{i}\right)\right]f\left(x\right)dx+\\ \int_{q_{h}+q_{i}}^{\infty }\left[p\left(q_{h}+q_{i}\right)-s\left(x-q_{h}-q_{i}\right)-w_{e}\left(q_{h}+q_{i}\right)\right]f\left(x\right)dx] \end{array}$$

The optimal order quantity of medical institutions under the repurchase contract can be obtained by calculating the derivatives *q*_*h*_ and *q*_*i*_, respectively:


$$\begin{array}{l} Q^{*}={q_{h}}^{*}+{q_{i}}^{*}=F^{-1}\left(\frac{w_{e}-p-s}{\gamma w_{e}-p-s}\right) \end{array}$$

### Revenue of pharmaceutical manufacturers under combined contract

Under this scenario, the expected revenue of the pharmaceutical manufacturer is:


(22) $$\begin{array}{l} E\left({\pi_{e}}^{m}\right)=\theta [w_{e}\text{ min}\left(q_{i},x\right)-s\text{ max}\left(x-q_{i},0\right)\\ +\left(v-\gamma w_{e}\right)\text{ max}\left(q_{i}-x,0\right)-w_{i}q_{i}]+\left(1-\theta \right)\\ {}[w_{e}\text{ min}\left(q_{h}+q_{i},x\right)-s\text{ max}\left(x-q_{i}-q_{h},0\right)\\ +\left(v-\gamma w_{e}\right)\text{ max}\left(q_{h}+q_{i}-x,0\right)-w_{i}q_{i}-w_{h}q_{h}] \end{array}$$

Rewrite the above formula in integral form as follows:


(23) $$\begin{array}{l} {\pi_{e}}^{m}=\theta \int_{0}^{q_{i}}\left[w_{e}x+\left(v-\gamma w_{e}\right)\left(q_{i}-x\right)-w_{i}q_{i}\right]f\left(x\right)dx\\ +\theta \int_{q_{i}}^{+\infty }\left[w_{e}q_{i}-s\left(x-q_{i}\right)-w_{i}q_{i}\right]\\ f\left(x\right)dx+\left(1-\theta \right)\int_{0}^{q_{h}+q_{i}}[w_{e}x+\left(v-\gamma w_{e}\right)\left(q_{h}+q_{i}-x\right)-w_{i}q_{i}\\ -w_{h}q_{h}]f\left(x\right)dx+\left(1-\theta \right)\int_{q_{h}+q_{i}}^{+\infty }[w_{e}(q_{h}\\ +q_{i})-s\left(x-q_{h}-q_{i}\right)-w_{i}q_{i}-w_{h}q_{h}]f\left(x\right)dx \end{array}$$

Similarly, we can get:


(24) $$\begin{array}{l} \frac{\partial \pi^{\prime }}{\partial q_{i}}=\left(v-\gamma w_{e}-w_{e}-s\right)\left[\left(1-\theta \right)F\left(q_{h}+q_{i}\right)+\theta F\left(q_{i}\right)\right]+w_{e}\\ \;+s-w_{i} \end{array}$$

(25) $$\begin{array}{l} \frac{\partial^{2}\pi^{\prime }}{\partial {q_{i}}^{2}}=\left(v-\gamma w_{e}-w_{e}-s\right)\left[\left(1-\theta \right)f\left(q_{h}+q_{i}\right)+\theta f\left(q_{i}\right)\right] \end{array}$$

From these assumptions, we can easily know that $\frac{\partial^{2}\pi^{\prime }}{\partial {q_{i}}^{2}}<0$ is constant, and the satisfying conditions of the optimal order quantity ${q_{i}}^{*}$ are obtained as follows:


(26) $$\begin{array}{l} \left(1-\theta \right)F\left(q_{h}+{q_{i}}^{*}\right)+\theta F\left({q_{i}}^{*}\right)=\frac{w_{i}-w_{e}-s}{\left(v-\gamma w_{e}-w_{e}-s\right)} \end{array}$$

### Revenue of pharmaceutical backup supplier under combined contract

The revenue obtained by the pharmaceutical manufacturer from the main supplier A of pharmaceutical raw materials is recorded as π_*e*1_, and the revenue obtained from the backup supplier B of pharmaceutical raw materials is recorded as π_*e*2_.


(27) $$\begin{array}{l} \pi_{e1}\left(q_{h}\right)=\left(1-\theta \right)[\int_{0}^{q_{h}}[w_{e}xf\left(x\right)dx+\int_{q_{h}}^{+\infty }[w_{e}q_{h}f\left(x\right)dx\\ +\int_{0}^{q_{h}}v\left(q_{h}-x\right)f\left(x\right)dx-\\ \int_{q_{h}}^{q_{h}+q_{1}}s\left(x-q_{h}\right)f\left(x\right)dx]-\theta \int_{q_{1}}^{+\infty }sxf\left(x\right)dx \end{array}$$

(28) $$\begin{array}{l} \pi_{e2}\left(q_{i}\right)={\pi_{e}}^{m}\left(q_{i}\right)-\pi_{e1}\left(q_{h}\right) \end{array}$$

With a revenue sharing factor κ, pharmaceutical manufacturers share their revenues from the backup supplier π_*e*2_ to the backup supplier. The revenue function of the backup supplier under the combination contract is obtained as follows:


(29) $$\begin{array}{l} {\pi_{i}}^{n}=\pi_{i}+\kappa \left({\pi_{e}}^{m}-\pi_{e1}\right) \end{array}$$

Bring Equations (9), (23), and (27) into the above formula and derive *q*_*i*_ to obtain:


(30) $$\begin{array}{l} \frac{\partial {\pi_{i}}^{n}}{\partial q_{i}}=\left[\left(v-w_{i}\right)+\kappa \left(v-\gamma w_{e}-w_{e}-s\right)\right][\left(1-\theta \right)F\left(q_{h}+q_{i}\right)\\ \;+\theta F\left(q_{i}\right)]+\kappa \left(w_{e}+s-w_{i}\right)+\left(w_{i}-c_{i}\right) \end{array}$$

(31) $$\begin{array}{l} \frac{\partial^{2}{\pi_{i}}^{n}}{\partial {q_{i}}^{2}}=\left[\left(v-w_{i}\right)+\kappa \left(v-\gamma w_{e}-w_{e}-s\right)\right][\left(1-\theta \right)f\left(q_{h}+q_{i}\right)\\ \;+\theta f\left(q_{i}\right)]<0 \end{array}$$

That is, after joining the revenue-sharing contract, the backup supplier has the maximum revenue and ${\pi_{i}}^{n}\left({q_{i}}^{n*}\right)$ meets the following formula:


(32) $$\begin{array}{l} \left(1-\theta \right)F\left(q_{h}+{q_{i}}^{n*}\right)+\theta F\left({q_{i}}^{n*}\right)=\frac{c_{i}-w_{i}-\kappa \left(w_{e}+s-w_{i}\right)}{\left(v-w_{i}\right)+\kappa \left(v-\gamma w_{e}-w_{e}-s\right)} \end{array}$$

Proposition: The income sharing coefficient κ should be valued at the interval


$$\begin{array}{l} \left[\frac{\pi_{i}\left({q_{i}}^{*}\right)-\pi_{i}\left({q_{i}}^{n*}\right)}{\pi_{e}\left({q_{i}}^{n*}\right)-\pi_{e1}\left(q_{h}\right)},\frac{\pi_{e}\left({q_{i}}^{n*}\right)-\pi_{e}\left({q_{i}}^{*}\right)}{\pi_{e}\left({q_{i}}^{n*}\right)-\pi_{e1}\left(q_{h}\right)}\right]. \end{array}$$

Proof: If the pharmaceutical manufacturer and the backup supplier sign a revenue-sharing contract to achieve coordination of the contract, it is necessary to ensure that after the pharmaceutical producer and backup supplier join the contract, their respective benefits are no less than under decentralized conditions.


(33) $$\begin{array}{l} {\pi_{e}}^{n}\left({q_{i}}^{n*}\right)=\pi_{e}\left({q_{i}}^{n*}\right)-\kappa \left[\pi_{e}\left({q_{i}}^{n*}\right)-\pi_{e1}\left(q_{h}\right)\right]\geq \pi_{e}\left({q_{i}}^{*}\right) \end{array}$$

(34) $$\begin{array}{l} {\pi_{i}}^{n}\left({q_{i}}^{n*}\right)=\pi_{i}\left({q_{i}}^{n*}\right)+\kappa \left[\pi_{e}\left({q_{i}}^{n*}\right)-\pi_{e1}\left(q_{h}\right)\right]\geq \pi_{i}\left({q_{i}}^{*}\right) \end{array}$$

Combining Equations (33) and (34), the value range of κ can be obtained as follows:


(35) $$\begin{array}{l} \frac{\pi_{i}\left({q_{i}}^{*}\right)-\pi_{i}\left({q_{i}}^{n*}\right)}{\pi_{e}\left({q_{i}}^{n*}\right)-\pi_{e1}\left(q_{h}\right)}\leq \kappa \leq \frac{\pi_{e}\left({q_{i}}^{n*}\right)-\pi_{e}\left({q_{i}}^{*}\right)}{\pi_{e}\left({q_{i}}^{n*}\right)-\pi_{e1}\left(q_{h}\right)} \end{array}$$

when κ is within the above range, the backup supplier's supply ${q_{i}}^{n*}$ is closer to the supply ${q_{i}}^{c*}$ under the centralized condition, which is much larger than the supply ${q_{i}}^{d*}$ under the decentralized condition. The revenues of backup suppliers, pharmaceutical manufacturers, and medical institutions are not less than their respective revenues under decentralized conditions, which shows that the combination contract based on buyback-benefit sharing can coordinate the pharmaceutical supply chain under an interruption crisis and achieve Pareto improvement.

## Results and discussion

To verify the effectiveness of the mathematical model built earlier, a correlation analysis of each parameter is performed. These parameters are chosen as follows:*c*_*i*_ = 10,*c*_*h*_ = 8,*w*_*i*_ = 20,*w*_*h*_ = 15,*w*_*e*_ = 25,*p* = 40,*s* = 20,*v* = 5, and the random market demand follows the distribution of *N*(100, 10^2^).

**Figure 2:**
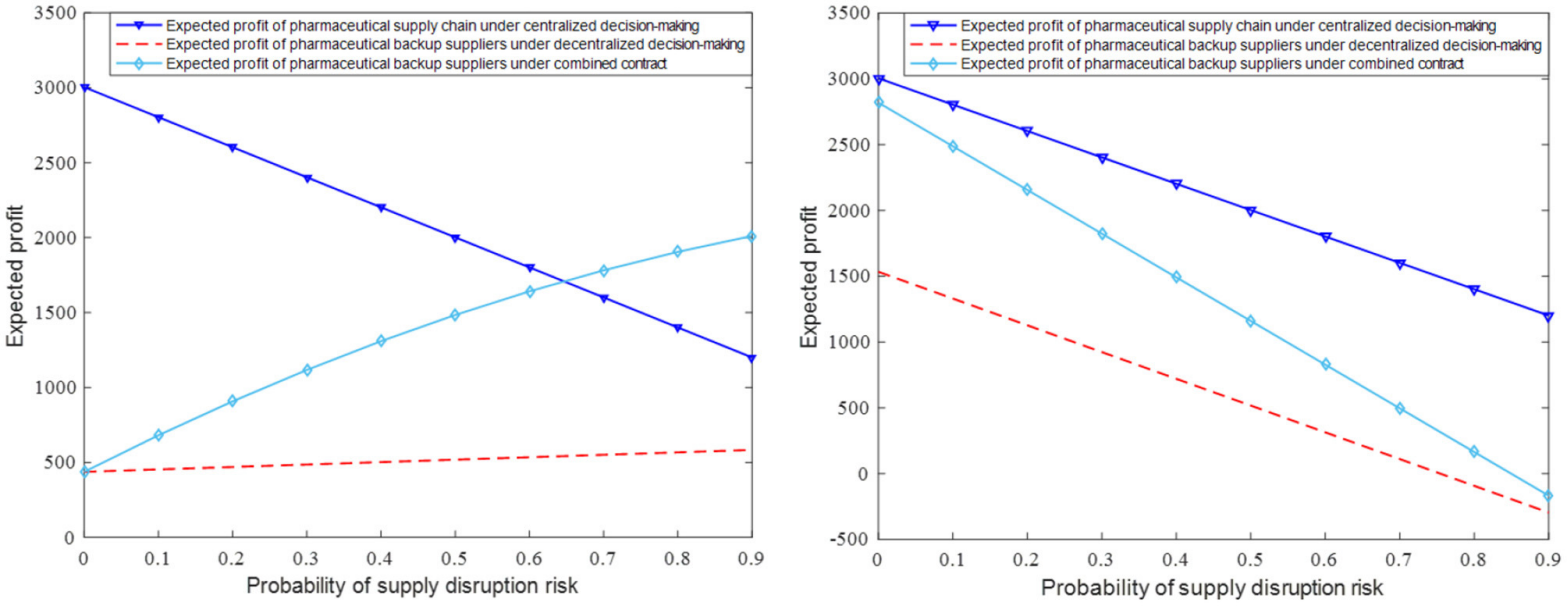
The impact of parameter θ on expected revenue of pharmaceutical backup suppliers and pharmaceutical manufacturer.

When γ = 0.91 and κ = 0.3, we obtain the expected revenue performance of the whole pharmaceutical supply chain and members under different scenes when the supply interruption risk probability is in the interval (0, 0.9), as shown in Figure 2. It can be seen that the expected revenues of pharmaceutical producers have decreased and those of the standby suppliers of pharmaceutical raw materials have increased as the probability of supply interruption has increased. This is due to the high risk of interruption from major suppliers and the increased procurement cooperation between pharmaceutical manufacturers and backup suppliers, which allows stable backup suppliers to be protected from supply interruptions and thus achieve higher returns. In addition, it can be found that the revenue when the drug manufacturer and the backup supplier sign the merger contract will be much higher than the revenue when the decentralized decision is made so that the pharmaceutical supply chain can be optimized and coordinated.

**Figure 3:**
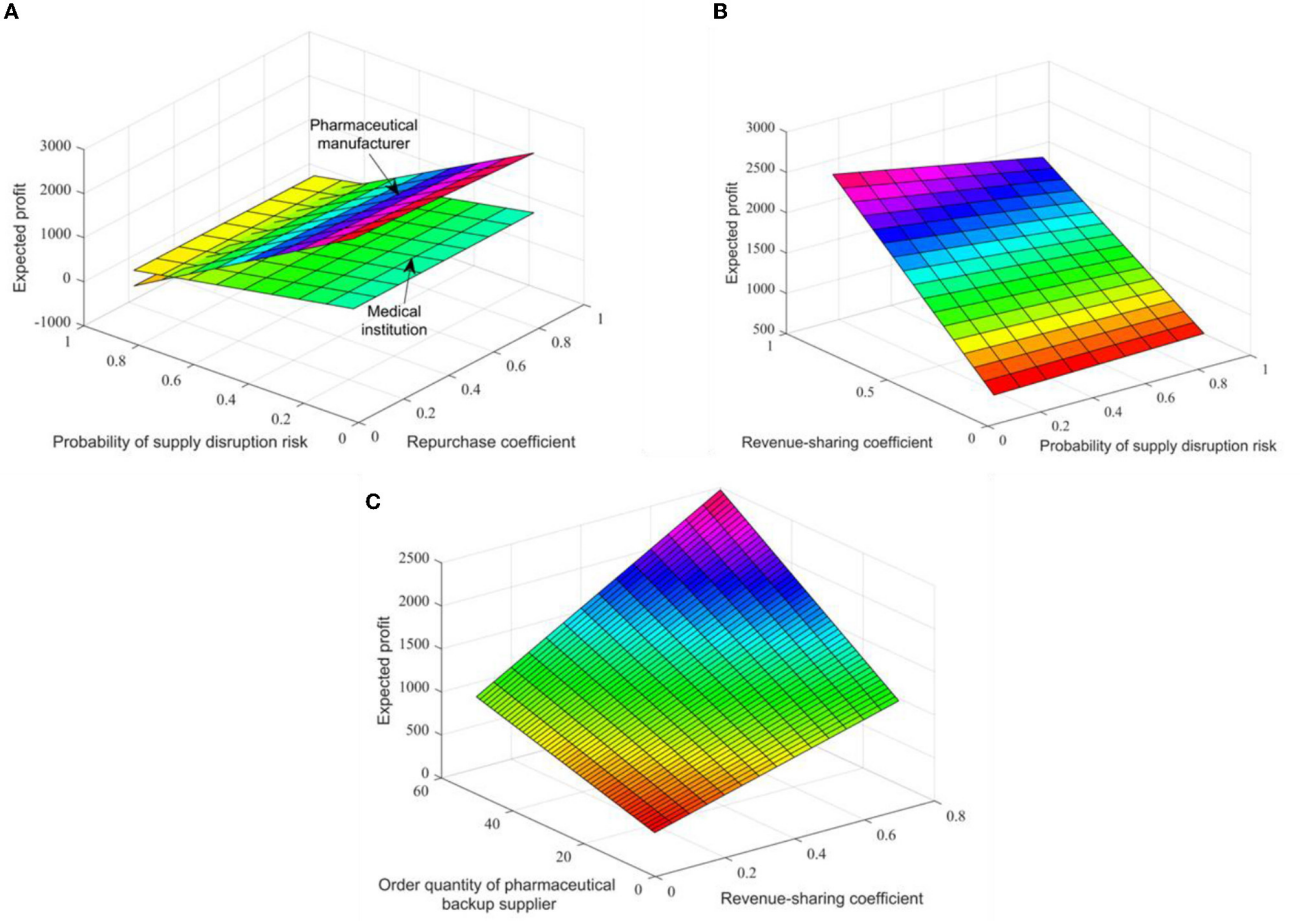
The impact of different contract parameters on the expected revenue of pharmaceutical supply chain members in figures **(A–C)**.

When *q*_*i*_ = 60 and *q*_*h*_ = 50, the impact of different contract parameter combinations (γ, κ, θ) on the expected revenue of the pharmaceutical supply chain members is shown in Figures 3A, B. In comparison, the repurchase coefficient has little effect on the expected revenue of pharmaceutical manufacturers and medical institutions. Pharmaceutical manufacturers' expected revenue slightly decreases with the increase of the repurchase coefficient, while medical institutions' expected revenues slightly increase. Figure 3B shows that with the increase of the coefficient of revenue sharing, the expected revenue of backup suppliers will also increase significantly. This is because even if pharmaceutical manufacturers increase the recovery price coefficient, the recovery price is still low, which can only make up for some losses in medical institutions. To encourage backup suppliers to supply, pharmaceutical manufacturers have obvious revenue advantages in signing revenue-sharing contracts.

Assuming that θ = 0.3, the changes in expected revenues and supply volume of pharmaceutical raw material backup suppliers within the range (0.1, 0.9) of income sharing coefficient are shown in Figure 3C. Thus, an increase in the revenue distribution factor will increase the expected revenues and supply of backup suppliers, that is, the number of orders placed by pharmaceutical companies. Practice shows that the combination contract can optimize the coordination of the pharmaceutical supply chain, and there is an optimal ordering strategy to make the pharmaceutical supply chain profitable to the level of centralized decision-making.

## Conclusion

In this study, we constructed a three-tiered supply chain composed of pharmaceutical raw material suppliers, pharmaceutical manufacturers, and institutions. Assuming that the market demand follows a random distribution, pharmaceutical manufacturers complete the production objectives and meet the needs of patients through a joint supply of a major supplier and a backup supplier. There is a large shortage cost in downstream medical institutions. Once the supply cannot meet the needs of patients, medical institutions will bear huge punishment costs and produce shortage losses. By introducing repurchase contracts between medical institutions and pharmaceutical manufacturers and introducing revenue-sharing contracts between backup suppliers and pharmaceutical manufacturers, it is proved that the corresponding combined contracts can increase the supply of backup suppliers and meet the needs of the pharmaceutical market, effectively respond to the supply interruption crisis of the main supplier, significantly improve the revenues of medical institutions and backup suppliers, and achieve the purpose of supply chain coordination. It is enlightening that pharmaceutical manufacturers need to cooperate with backup suppliers and medical institutions in a wider field and at a higher level in practice to reduce the risk of supply interruption of the main suppliers.

In addition, this paper has the following shortcomings: generally, in the actual operation of the pharmaceutical supply chain, a pharmaceutical manufacturer may articulate more than one pharmaceutical organization, or a pharmaceutical raw material supplier serves more than one pharmaceutical manufacturer, and this study only considers the pharmaceutical supply chain members. Therefore, in the future, we should consider studying the sustainable development model of a complex supply chain ecosystem with multiple levels and multiple subjects.

## Data availability statement

The original contributions presented in the study are included in the article/supplementary material, further inquiries can be directed to the corresponding author.

## Author contributions

GF conceived the model and project. ZZ analyzed results and wrote the manuscript. All authors read, edited, and approved the final manuscript.
